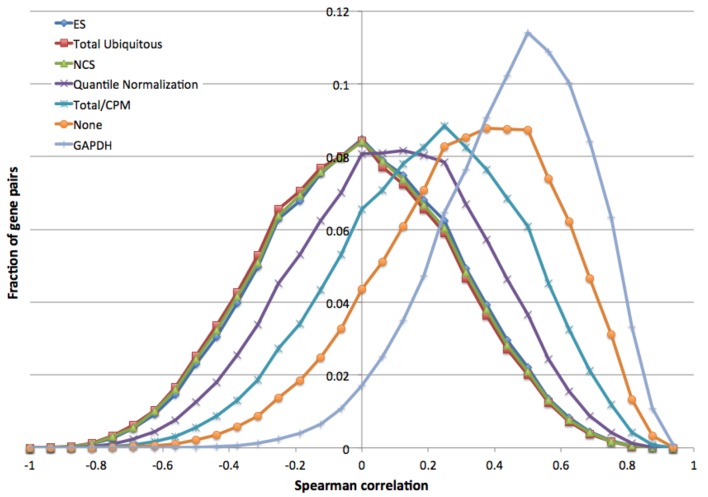# Correction: Optimal Scaling of Digital Transcriptomes

**DOI:** 10.1371/annotation/8b05a9ab-c8ad-4276-a851-1e265055fb65

**Published:** 2014-01-08

**Authors:** Gustavo Glusman, Juan Caballero, Max Robinson, Burak Kutlu, Leroy Hood

A funding organization was incorrectly omitted from the Funding Statement. The Funding Statement should read: "This work was supported by contract W911SR-09-C-0062 from Edgewood Contracting Division, US Army, and by the University of Luxembourg - Institute for Systems Biology Program. The funders had no role in study design, data collection and analysis, decision to publish, or preparation of the manuscript."

In addition, Figures 2B, 3, and 4 were incorrectly switched. The image currently appearing as Figure 3 is Figure 2B and belongs with the title and legend for Figure 2, the image currently appearing as Figure 4 belongs with the title and legend for Figure 3, and the image currently appearing as Figure 5 belongs with the title and legend for Figure 4.

In addition, the correct version of Figure 5 is missing. Please see the correct version of Figure 5 here: 

**Figure:**