# Correction: Optimal Scaling of Digital Transcriptomes

**DOI:** 10.1371/journal.pone.0093244

**Published:** 2014-03-18

**Authors:** The PLOS ONE Staff

## Notice of Republication

This article was republished on February 28, 2014, to replace an incorrectly published version. The new version includes all figures in the correct order and addresses the figure issues noted in the previous Formal Correction. The originally published, uncorrected article and the republished, corrected article are provided here for reference.

## Supporting Information

File S1 Originally published, uncorrected article.
(PDF)

File S2 Republished, corrected article.
(PDF)
