# Supplementary figures and images for: Optimal Scaling of Digital Transcriptomes
Source: PLoS One. 2013 Nov 6;8(11):e77885. doi: 10.1371/journal.pone.0077885 (PMC3819321; doi:10.1371/journal.pone.0077885)

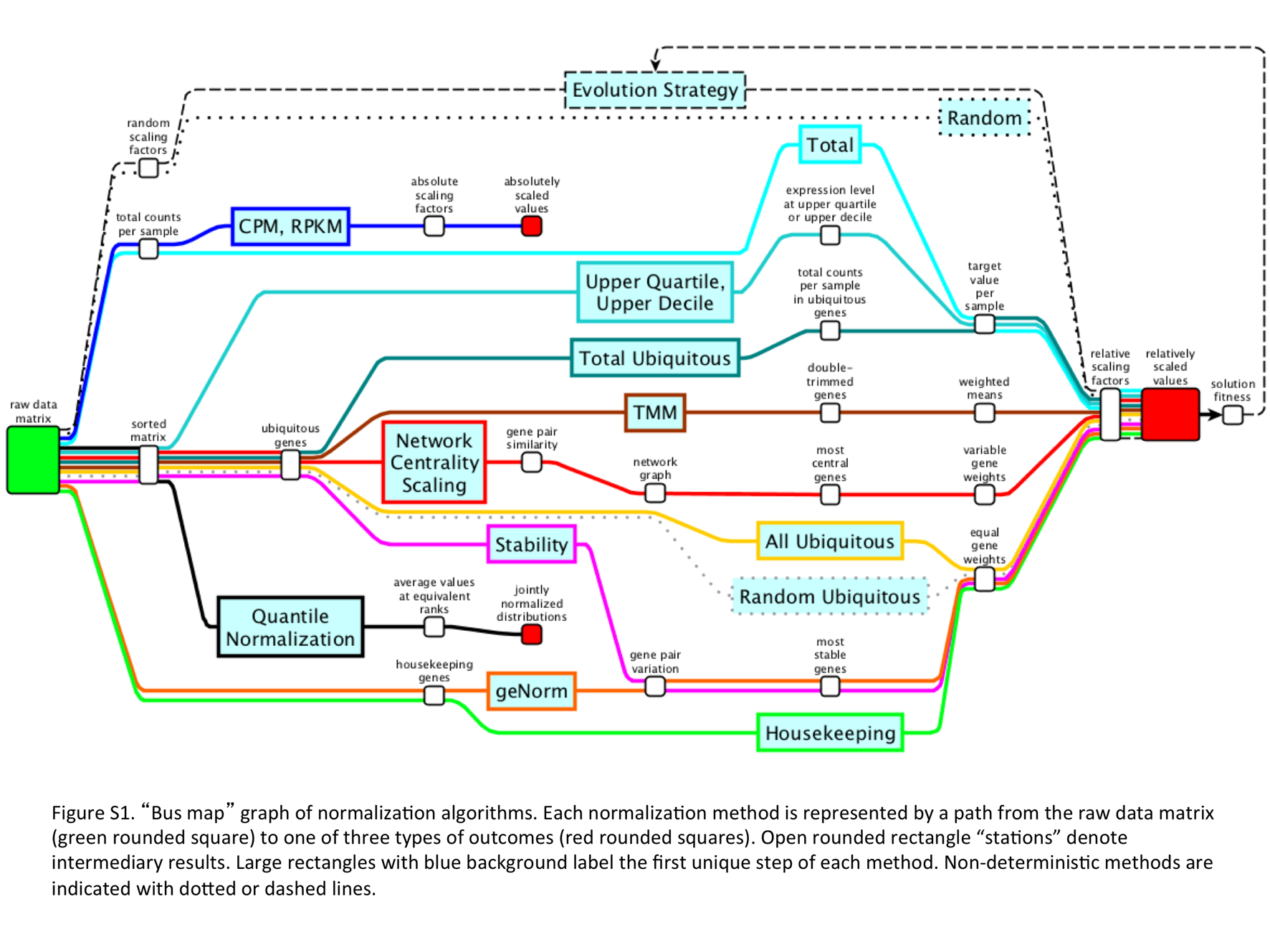

Supplement: Figure S1 — “Bus map” graph of normalization algorithms. Each normalization method is represented by a path from the raw data matrix (green rounded square) to one of three types of outcomes (red rounded squares). Open rounded rectangle “stations” denote intermediary results. Large rectangles with blue background label the first unique step of each method. Non-deterministic methods are indicated with dotted or dashed lines. (TIFF) [file pone.0077885.s001.tiff]

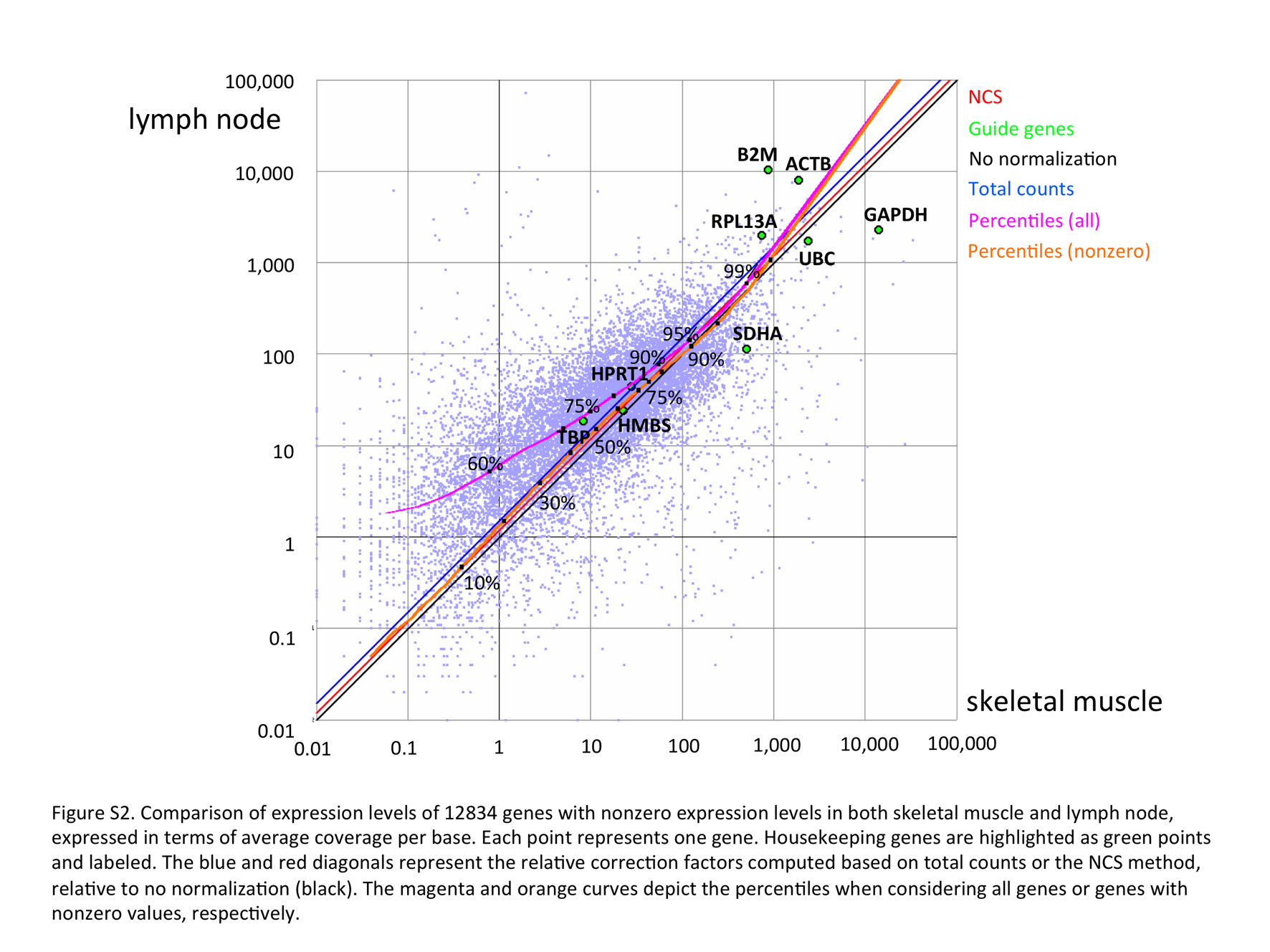

Supplement: Figure S2 — Comparison of expression levels of 12834 genes with nonzero expression levels in both skeletal muscle and lymph node, expressed in terms of average coverage per base. Each point represents one gene. Housekeeping genes are highlighted as green points and labeled. The blue and red diagonals represent the relative correction factors computed based on total counts or the NCS method, relative to no normalization (black). The magenta and orange curves depict the percentiles when considering all genes or genes with nonzero values, respectively. (TIFF) [file pone.0077885.s002.tiff]

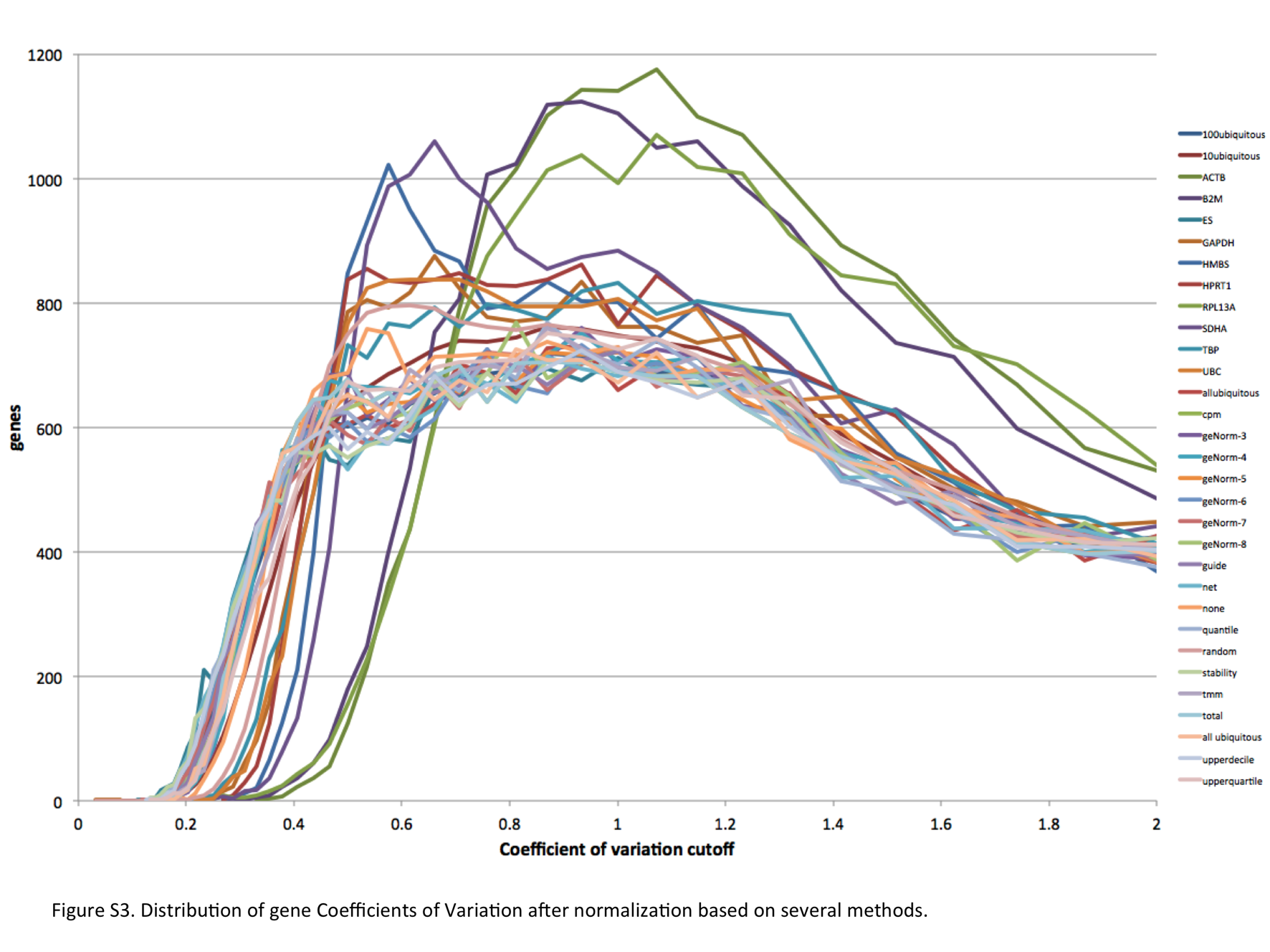

Supplement: Figure S3 — Distribution of gene Coefficients of Variation after normalization based on several methods. (TIFF) [file pone.0077885.s003.tiff]

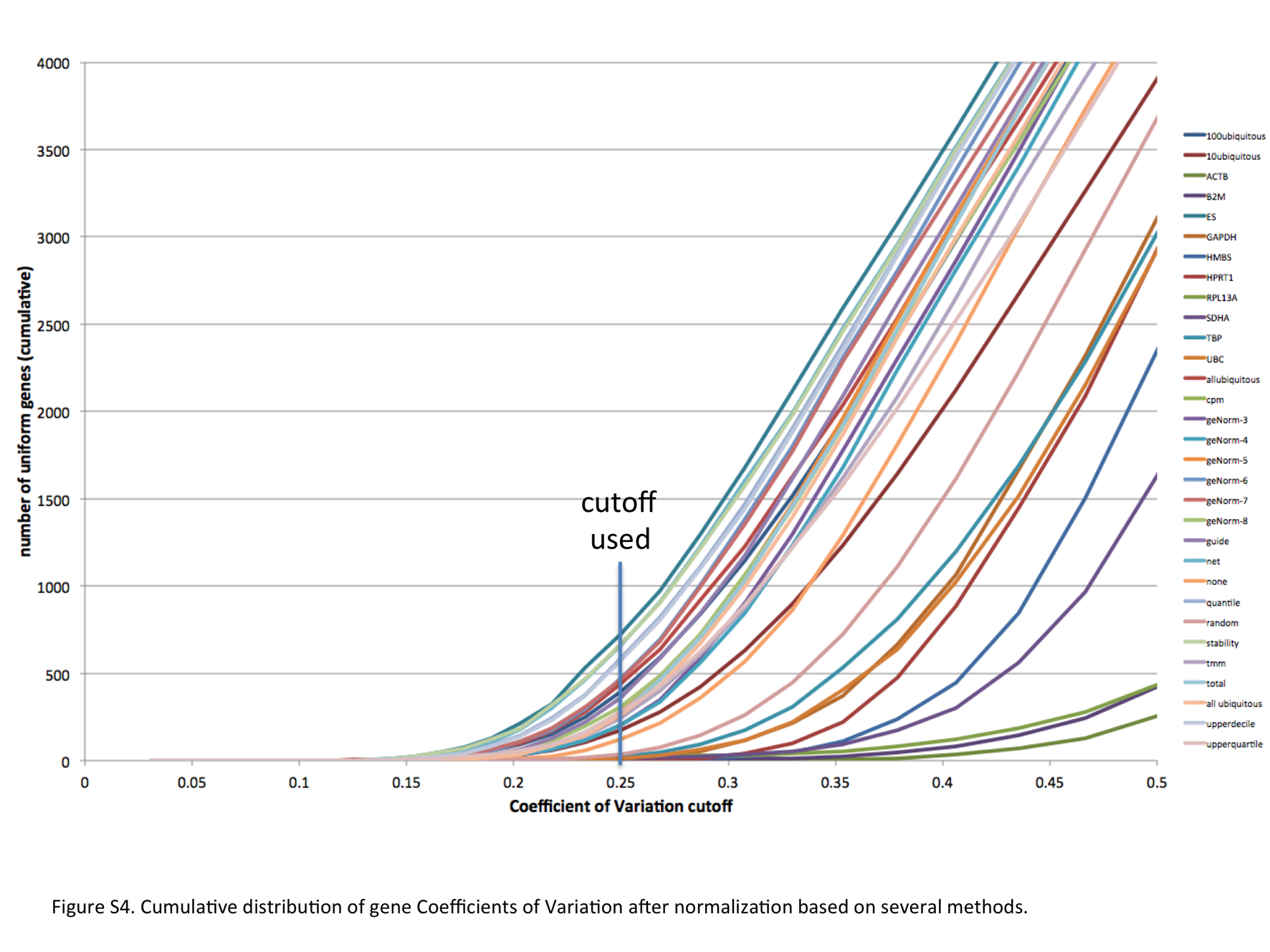

Supplement: Figure S4 — Cumulative distribution of gene Coefficients of Variation after normalization based on several methods. (TIFF) [file pone.0077885.s004.tiff]

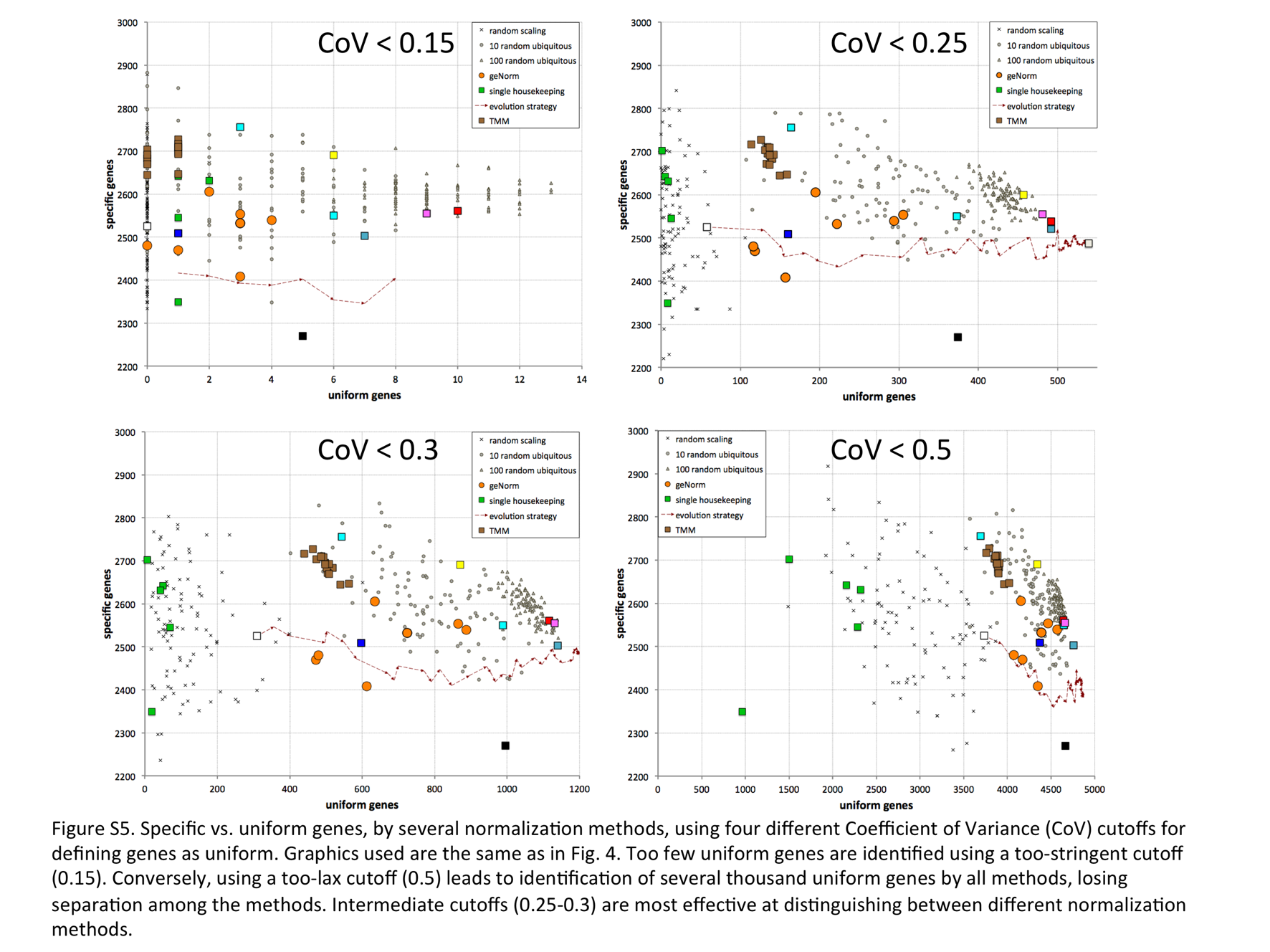

Supplement: Figure S5 — Specific vs. uniform genes, by several normalization methods, using four different Coefficient of Variance (CoV) cutoffs for defining genes as uniform. Graphics used are the same as in Fig. 4. Too few uniform genes are identified using a too-stringent cutoff (0.15). Conversely, using a too-lax cutoff (0.5) leads to identification of several thousand uniform genes by all methods, losing separation among the methods. Intermediate cutoffs (0.25–0.3) are most effective at distinguishing between different normalization methods. (TIFF) [file pone.0077885.s005.tiff]

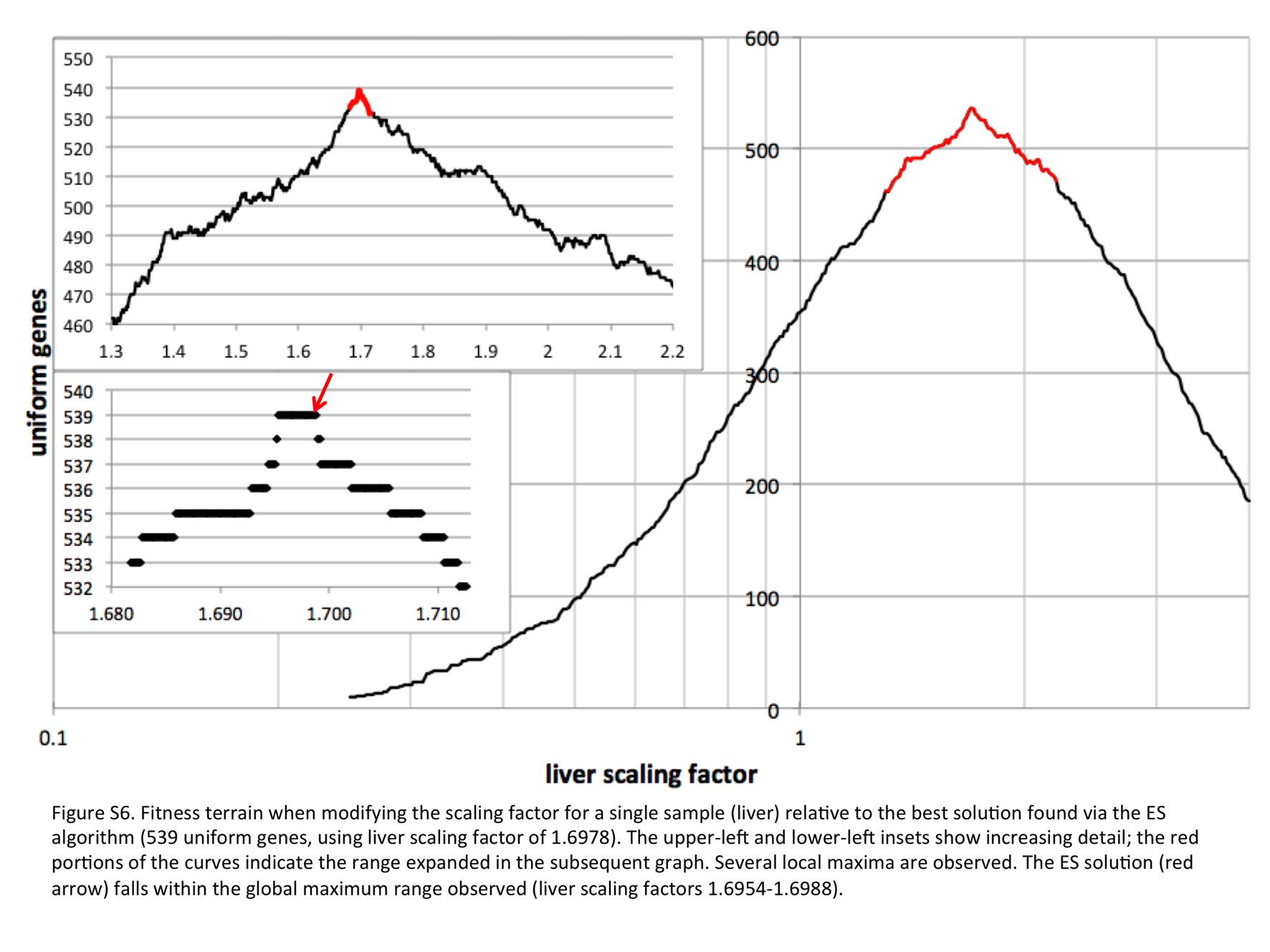

Supplement: Figure S6 — Fitness terrain when modifying the scaling factor for a single sample (liver) relative to the best solution found via the ES algorithm (539 uniform genes, using liver scaling factor of 1.6978). The upper-left and lower-left insets show increasing detail; the red portions of the curves indicate the range expanded in the subsequent graph. Several local maxima are observed. The ES solution (red arrow) falls within the global maximum range observed (liver scaling factors 1.6954–1.6988). (TIFF) [file pone.0077885.s006.tiff]

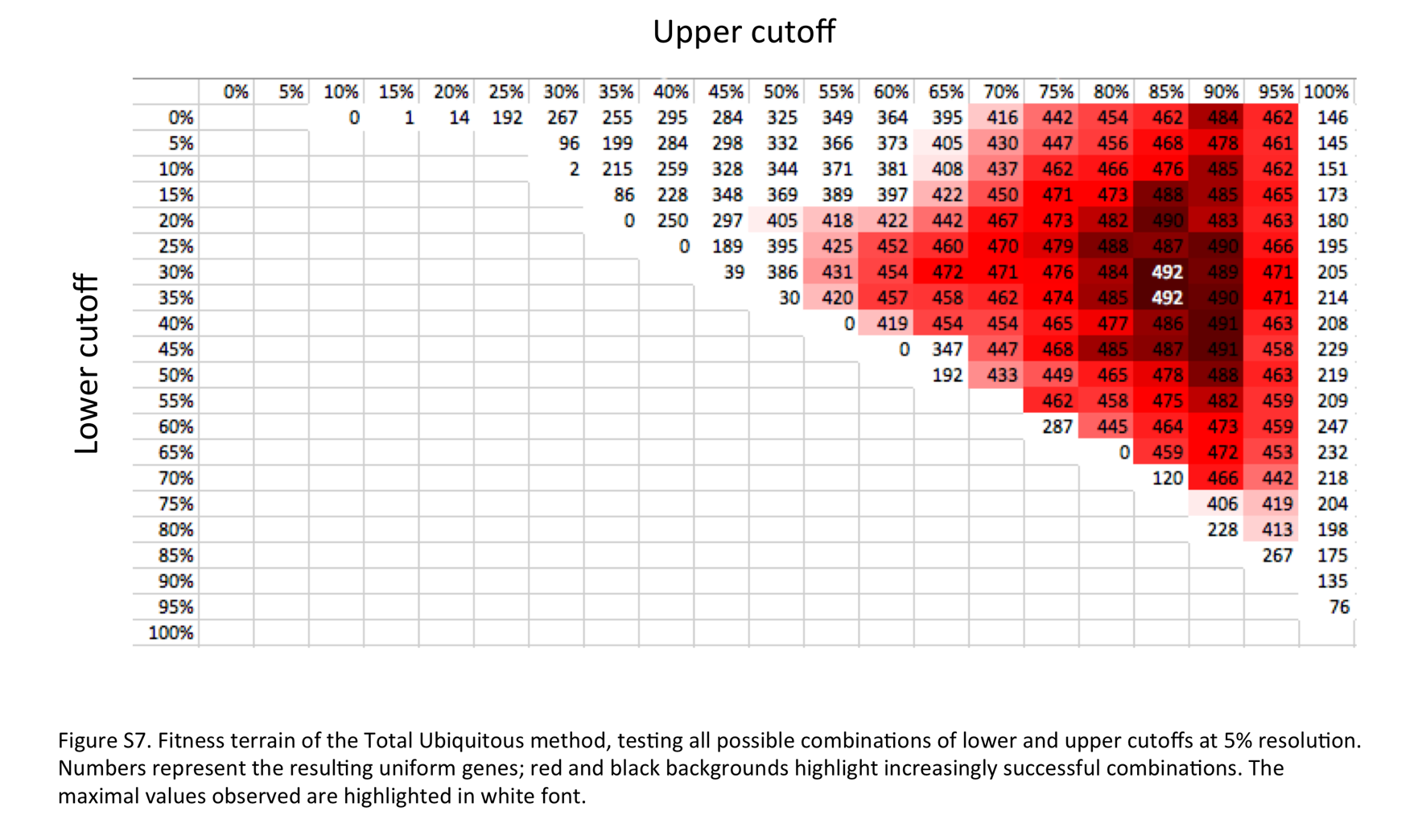

Supplement: Figure S7 — Fitness terrain of the Total Ubiquitous method, testing all possible combinations of lower and upper cutoffs at 5% resolution. Numbers represent the resulting uniform genes; red and black backgrounds highlight increasingly successful combinations. The maximal values observed are highlighted in white font. (TIFF) [file pone.0077885.s007.tiff]
