# Supplementary material for: Correction: Optimal Scaling of Digital Transcriptomes
Source: PLoS One. 2014 Mar 18;9(3):e93244. doi: 10.1371/journal.pone.0093244 (PMC3958558; doi:10.1371/journal.pone.0093244)
Supplement: File S2 — Republished, corrected article. (PDF) [file pone.0093244.s002.pdf]

PLOS ONE

Optimal Scaling of Digital Transcriptomes

Gustavo Glusman[1]*, Juan Caballero[2], Max Robinson[1], Burak Kutlu[1], Leroy Hood[1]

1 Institute for Systems Biology, Seattle, Washington, United States of America, 2 Facultad de Ingeniería, Universidad Autónoma de Querétaro, Querétaro, México

Abstract

Deep sequencing of transcriptomes has become an indispensable tool for biology, enabling expression levels for thousands of genes to be compared across multiple samples. Since transcript counts scale with sequencing depth, counts from different samples must be normalized to a common scale prior to comparison. We analyzed fifteen existing and novel algorithms for normalizing transcript counts, and evaluated the effectiveness of the resulting normalizations. For this purpose we defined two novel and mutually independent metrics: (1) the number of "uniform" genes (genes whose normalized expression levels have a sufficiently low coefficient of variation), and (2) low Spearman correlation between normalized expression profiles of gene pairs. We also define four novel algorithms, one of which explicitly maximizes the number of uniform genes, and compared the performance of all fifteen algorithms. The two most commonly used methods (scaling to a fixed total value, or equalizing the expression of certain 'housekeeping' genes) yielded particularly poor results, surpassed even by normalization based on randomly selected gene sets. Conversely, seven of the algorithms approached what appears to be optimal normalization. Three of these algorithms rely on the identification of "ubiquitous" genes: genes expressed in all the samples studied, but never at very high or very low levels. We demonstrate that these include a "core" of genes expressed in many tissues in a mutually consistent pattern, which is suitable for use as an internal normalization guide. The new methods yield robustly normalized expression values, which is a prerequisite for the identification of differentially expressed and tissue-specific genes as potential biomarkers.

Citation: Glusman G, Caballero J, Robinson M, Kutlu B, Hood L (2013) Optimal Scaling of Digital Transcriptomes. PLoS ONE 8(11): e77885. doi:10.1371/journal.pone.0077885

Editor: I. King Jordan, Georgia Institute of Technology, United States of America

Received August 28, 2013; **Accepted** September 9, 2013; **Published** November 6, 2013

Copyright: © 2013 Glusman et al. This is an open-access article distributed under the terms of the Creative Commons Attribution License, which permits unrestricted use, distribution, and reproduction in any medium, provided the original author and source are credited.

Funding: This work was supported by contract W911SR-09-C-0062 from Edgewood Contracting Division, US Army. The funders had no role in study design, data collection and analysis, decision to publish, or preparation of the manuscript.

Competing Interests: The authors have declared that no competing interests exist.

* E-mail: Gustavo@SystemsBiology.org

Introduction

Modern sequencing technologies have enabled measurement of gene expression by "digital transcript counting". Transcript counting has a number of compelling advantages, including high sensitivity and the ability to discover previously unknown transcripts [1]. Transcript counting started with the original Serial Analysis of Gene Expression method (SAGE) [2], gained momentum with Massively Parallel Signature Sequencing (MPSS) [3] and is coming to maturity with the application of "next generation" high throughput sequencing technologies. In particular, the modern RNA-seq technology [4] sequences the full extent of each transcript, and therefore has the added advantage of being able to characterize alternative splice forms for the same gene [5]. Alternative splicing can be cell type-specific, tissue-specific, sex-specific and lineage-specific [6]. More recently, SAGE-Seq was described [7], applying next-generation sequencing to obtaining SAGE-like data.

After correcting for gene length [4,8] and for composition and sequence-specific effects, particularly GC-content [9,10], the expression levels of different genes and transcripts within the same sample are directly comparable. On the other hand, the comparison of gene expression levels across samples (to identify differentially expressed and tissue-specific genes) necessitates normalization of counts for each sample to a common scale. Digital transcript counting methods measure transcript expression as observation of a transcript k times at a sequencing depth of N sequenced transcripts, and thus relative to the total expressed content of the sample. Thus differential expression of any transcript between two samples implicitly affects the measurement of all transcripts, complicating the process of determining the relative scale of counts from different samples. When attempting to identify differentially expressed genes from transcript count data, incorrect normalization may therefore lead to both false positives and false negatives [11,12].

Many count normalization methods have been proposed to date (Fig. 1), most frequently by effectively determining a single sample-specific scaling factor [11,12]. The most commonly used method normalizes expression values to the total number of reads observed in each sample: gene expression values are thus expressed in terms of "transcripts per million" or "counts per million" (CPM); for RNA-seq, the equivalent measure is "reads per kb per million" (RPKM) [4]. This method implicitly assumes that all cell types express total RNA to equivalent levels, and therefore that CPM/RPKM values are directly comparable across samples [13]. This method has the advantage of simplicity as samples can be normalized independently, but there is no *a priori* reason to assume that the total RNA content should be constant across cell types [14,15]. Importantly, the results are sensitive to the expression levels of highly expressed genes [16]: since much sequencing output is spent on them, the presence of a few, highly-expressed tissue-specific genes can significantly lower the CPM values for all other genes in the same sample, often leading to the wrong conclusion that the latter are "down-regulated".

A way to reduce sensitivity to highly expressed genes is to choose a scaling factor using a rank statistic; the median expression

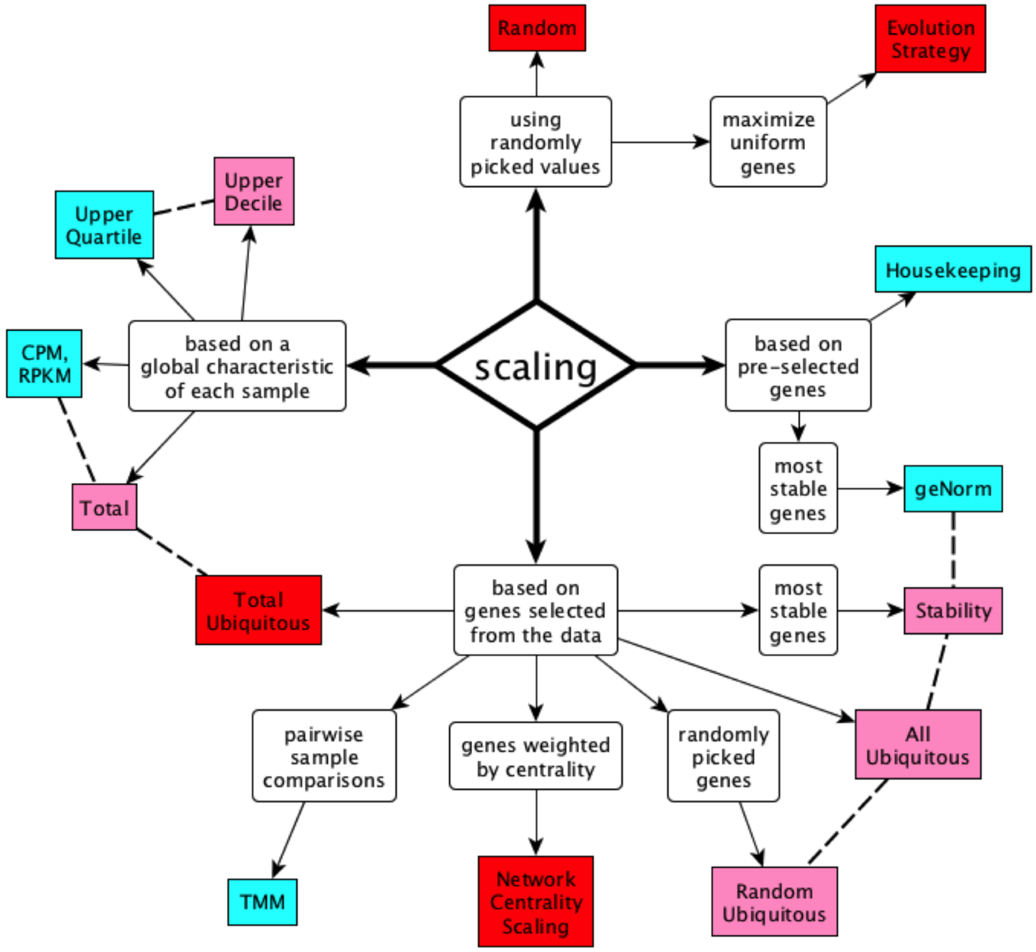

Figure 1. Conceptual taxonomy of scaling methods. Blue: published methods. Pink: variations on published methods. Red: novel methods. Dashed lines connect related methods.
doi:10.1371/journal.pone.0077885.g001

value is commonly used for normalization of microarray data [11,17]. Due to the preponderance of zero and low-count genes in digital transcriptomes, the median count may be zero or reflect stochastic sampling of rare transcripts and thus be an inaccurate measure of overall scale, but the scaling factor can be estimated from a higher rank statistic, such as the upper quartile [16]; however in our experience even the upper quartile value may be unsuitable, depending on the sparseness of the expression data matrix. Alternatively, it is possible to normalize samples quantile by quantile or rank by rank, rescaling relative expression levels within each sample to follow a common distribution [18]. This nonlinear method cancels global biases between samples, but destroys the inherently linear relationship between transcript counts and expression level within each sample and reduces power to detect differential expression [19]. Conditional quantile normalization improves the precision of this method by removing first systematic biases caused by GC-content and other deterministic features [20].

A different class of normalization methods relies on the expression level of a subset of the genes to "guide" the normalization. It is often assumed that the expression levels of certain "housekeeping" genes (e.g. *GAPDH*, *ACTB*) are constant across cell types, and can therefore be used individually as an internal normalization tool [14]. This assumption has been shown to be invalid in various scenarios, leading to incorrect results [21]. More elaborate methods in this class rely on minimizing the sample-to-sample variance of not just one, but a small set of guide genes [22], or estimate scaling factors from the trimmed mean of between-sample log-ratios for each gene, under the assumption that the majority of genes are not differentially expressed [12]. Finally, spiked-in standards have been used as an exogenous reference for determining the correct scaling factors for normalization [23]. This method has the advantage of being able to expose global changes in total RNA production [15] but are not yet in widespread use, and significant legacy data exist without spiked-in standards.

Each normalization method produces its own set of "corrected" expression values, leading to the identification of different, and usually conflicting, lists of differentially expressed genes. There is thus need to evaluate the relative success of different normalization protocols. Methods for validating normalization factors have relied on two kinds of metrics: performance in downstream analyses, and analysis of sample-to-sample variance remaining after normalization.

Results from qRT-PCR experiments (and earlier, Northern blots) have been used as a "gold standard" to determine the differential expression status of genes, and thus to evaluate normalization success [8,16]. False positive and false negative rates have also been assessed in simulation experiments, where the set of differentially expressed genes is specified in the simulation [12, Dillies, 2012 #41]. A more complex evaluation assesses the

ability of sets of normalized data to yield gene regulatory networks that translate between data sets [24].

Under the assumption that most genes are not differentially expressed, normalization is expected to reduce overall sample-to-sample variation in gene expression. Post-normalization variation has thus been used to evaluate success, in various ways: at the level of the individual housekeeping gene [22], by correlating one housekeeping gene to several others [14], and by computing the average coefficient of variation across all genes [18].

We present insights derived from the study of many normalization algorithms, including novel methods, with an emphasis on data driven approaches. We also present a framework for evaluating how successful normalization methods are at rendering the gene expression levels comparable across samples, without relying on specific applications (e.g., identifying differentially expressed genes). Our evaluation methods are entirely data driven and do not require "gold standard" RT-PCR data or simulations. We demonstrate that some algorithms independently produce very similar solutions, which are particularly successful and very different from that resulting from standard normalization methods. These results have deep implications for studies attempting to identify differentially expressed genes from RNA-seq data.

Materials and Methods

RNA-seq Analysis in Human Tissues

We obtained from Illumina, Inc. a set of 16 raw RNA-seq datasets (75 bp, single-end reads) from the following human tissues: adipose (HCT20158), adrenal (HCT20159), brain (HCT20160), breast (HCT20161), colon (HCT20162), kidney (HCT20142), heart (HCT20143), liver (HCT20144), lung (HCT20145), lymph node (HCT20146), prostate (HCT20147), skeletal muscle (HCT20148), white blood cells (HCT20149), ovary (HCT20150), testes (HCT20151) and thyroid (HCT20152). This data set is collectively referred to as BodyMap 2.0, and is available from the Gene Expression Omnibus as series GSE30611. To maximize mapped fraction in the presence of genetic variation, we aligned the sequence reads to the human reference genome (version hg19/GRCh37) using Blat v34 [25] with default parameters. We used custom Perl scripts to remove reads that map to more than 10 different genomic positions, to select the best alignment for each read, and to convert the output to SAM format. Blat is a more sensitive (and more computationally expensive) algorithm than the short read mappers typically used to map RNA-seq reads, and it can directly model long gaps corresponding to introns. Compared to TopHat/Bowtie [26], Blat mapped an additional 7.94% +/−2.3% of the total reads in each sample (from a sample average of 89.2% to 97.2%). Uniquely mapped reads increased by 8.85% +/−2.05% (from a sample average of 82.3% to 91.1%). We then assembled the mapped reads and correlated them with annotated transcripts (Ensembl Gene Models GRCh37.62) using Cufflinks v1.0.3 [27], keeping only reads uniquely mapped inside exonic regions of known genes, correcting for GC content and multiple mapping, and ignoring novel isoforms and pseudogenes, rRNAs and mitochondrial sequences (Cufflinks parameters: -b -G -u -M). We used custom Perl scripts to parse the Cufflinks output files and to summarize the expression levels of 133,810 observed transcripts as a table, using the "coverage" column to report the average fold coverage per transcribed base, avoiding the additional standard normalization to million reads (RPKM). Finally, we computed from these values the average fold coverage per transcribed base for each of 29,665 genes, as the average of its various transcripts, weighted by transcript length. Nearly one third of the genes (9,588, 32.2%) were observed in all samples.

Housekeeping Genes

We considered the ten housekeeping genes used in the geNorm study [22], namely: *ACTB, B2M, GAPDH, HMBS, HPRT1, RPL13A, SDHA, TBP, UBC* and *YWHAZ*. All of these had nonzero expression values in all 16 BodyMap 2.0 samples except for *YWHAZ* (observed in 9 out of 16 samples), which we therefore excluded from our study.

Definition of Ubiquitous Genes

Following the lead of Robinson and Oshlack [12], we identify for each sample a trimmed set of genes by 1) excluding genes with zero values, 2) sorting the non-zero genes by expression level in that sample, and 3) removing the upper and lower ends of the sample-specific expression distribution. To select the upper and lower cutoffs, we tested all possible combinations of lower and upper cutoffs at 5% resolution, and computed the number of resulting uniform genes for each combination (Fig. S7). This fitness terrain analysis shows that the upper cutoff is robust in the range of 70%–95%, while the lower cutoff can range from 0% to 60%. We retain genes from the 30th to 85th percentiles: these cutoffs maximize the number of uniform genes.

We define the set of "ubiquitous genes" as the intersection of the trimmed sets of all samples being considered. In other words, for a gene to be considered ubiquitous, it has to be expressed with nonzero values in all samples, and furthermore it cannot be in the top 15% or bottom 30% in any of the samples. Thus, by definition the set of "ubiquitous genes" excludes most if not all differentially expressed genes. This trimming method therefore implements Kadota's normalization strategy [28]. Using this definition and these cutoffs, we identified 2,507 ubiquitous genes in the 16-sample Illumina BodyMap 2.0 data set.

Some methods (e.g. Stability and NCS) are less affected by extremely expressed genes, and benefit from having a wider pool of options to select from. For these, we used the more inclusive cutoffs of Robinson and Oshlack [12], namely 5% from each end of the distribution. Using these cutoffs, we retained 6944 genes from the 16-sample Illumina BodyMap 2.0 data set.

Depending on the data set, it is possible that the resulting set of ubiquitous genes might be very small, or even empty. In such cases, we expand the definition to include genes that are included in the trimmed set of most samples (e.g., at least 80% of them). Conversely, if the set of ubiquitous genes is too large (e.g., for methods performing pairwise comparisons) or becomes too large after relaxing the inclusion cutoff, it is possible to select a subset of the ubiquitous genes by keeping those with higher expression values.

Definition of Specific Genes

We defined genes *specific* to a sample as those with a positive value for Jongeneel's specificity measure [29], i.e., the gene was observed in one sample at a level higher than the sum of all other samples combined. We further require that the gene be observed in at least half of the samples.

Computation of Relative Scaling Factors

Given a set of sample-specific scaling factors f_k, we compute an adjusted set f_k that maintains the global scale of the data set, by applying the constraint that the product of the values in the

adjusted set should be equal to 1: $f_k' = \frac{f_k}{2^{scale}}$, where $scale = \frac{\sum_k \log_2 f_k}{N}$ and N is the number of samples.

Decorrelation Analysis

Our second metric for qualifying the success of normalization methods is based on minimizing the correlation between the normalized expression profiles of gene pairs. To compute this, we analyzed the distribution of gene pair correlations of sample rankings, in three steps, as follows.

1) For each gene, independently of other genes, we sorted the samples based on the gene's expression levels. Each gene thus suggests a ranking of the samples. The ranking may change according to the normalization applied.
2) We compared ubiquitous genes in a pairwise fashion: for each pair of genes, we computed the Spearman correlation between the sample rankings suggested by each of the genes. High correlation values may reflect similarity in expression profiles of functionally related genes, but in the vast majority of the cases, correlation is inadvertently caused by distorted expression values. As an example of this effect, consider the gene expression values in testes vs. liver (Fig. 2A) as scaled by the "Total Counts" method (blue diagonal in the figure). For most genes in this comparison, the expression level in testes is higher than that observed in liver; most gene pairs would therefore rank the "testes" sample over the "liver" sample, leading to high Spearman correlation values. When considering the relative scaling of the two samples produced by the NCS method (red diagonal in the figure), approximately half the gene pairs rank "testes" over "liver", while the other half rank "liver" over "testes", leading to much lower Spearman correlation values.

For typical data sets, the number of gene pairs is too large; we therefore randomly selected a large sample of 100,000 gene pairs to characterize the distribution.

3) We summarized the results by computing the average correlation as the characteristic value of the distribution. We interpreted higher average correlations as indicative of less successful normalization, and average values near zero as denoting full decorrelation. We obtained similar results by using the median value.

Full List of Normalization Methods Implemented

We implemented six published normalization methods as well as some variations on them:

1) **CPM/RPKM** [4,13]. This method scales each sample to the same total count (one million). It differs from all other methods in that samples are scaled independently of each other.

Variant 1a) **Total Counts**. Instead of a fixed count, it scales each sample to the average total count per sample (equivalently: to the total number of reads in the entire data set, divided by the number of samples).

2) **Upper Quartile** [16]. This method scales the expression level at the 75th percentile in each sample to the average across all samples.

Variant 2a) **Upper Decile**. Scales the expression level at the 90th percentile in each sample to the average across all samples.

3) **Housekeeping** [14]. This method scales samples to equalize the observed expression level of a single, pre-selected guide gene. We considered nine known housekeeping genes: *HPRT, ACTB, GAPDH, TBP, UBC, SDHA, RPL13A, B2M* and *HMBS.*
4) **geNorm**. This method scales each sample to equalize the geometric mean of the expression levels of 3 to 9 control genes, selected among housekeeping genes (see 3 above) by a "gene stability" measure [22].

Variant 4a) **Stability**. Scales to equalize the geometric mean of the expression levels of the hundred most stable ubiquitous genes (using 5% trimming).

Variant 4b) **All Ubiquitous**. Scales using the geometric mean of expression levels of all ubiquitous genes.

Variant 4c) **Random Ubiquitous**. Scales using the geometric mean of the expression levels of n randomly-picked ubiquitous genes. We examined both n = 10 and n = 100, corresponding to the typical number of genes considered in the geNorm and NCS methods, respectively.

5) **Trimmed Mean of M-values (TMM)** [12]. Letting F_{gk} denote the fraction of transcript counts for gene g in sample k, this method analyzes each pair of samples as follows. After excluding genes that are not expressed in both samples, the genes with the top and bottom 5% geometric mean expression level $A_g = \log(F_{gi}) + \log(F_{gj})$ are trimmed; the asymptotic variance is also estimated as a function of A_g. Of the remaining genes, the genes with the top and bottom 30% log-expression ratio $M_g = \log(F_{gi})$ - $\log(F_{gj})$ are also trimmed. The log-ratio of the normalization constants for the two samples is then estimated as a weighted average of M_g over the remaining genes, using the inverse of the asymptotic variance corresponding to A_g as weights.
6) **Quantile Normalization** [18]. This nonlinear method makes the expression level distributions identical for all samples. In our implementation and as described [18], in each sample the normalized expression at each rank (not quantile) is set to the average at that rank across all samples.

We also implemented the following novel algorithms:

7) **Random**. A mock normalization method that scales each sample by a randomly picked factor 2^r, with r drawn uniformly in the range $[-0.5, 0.5)$. Thus, normalization factors were limited to 0.707 to 1.414.
8) **Total Ubiquitous**. Scales each sample to equalize the total counts for ubiquitous genes to the same value, the average across all samples.
9) **Network Centrality Scaling**. As detailed below.
10) **Evolution Strategy**. As detailed below.

Network Centrality Scaling

We developed a novel scaling algorithm based on network centrality analysis. Our method involves five computational stages:

Stage 1: Selection of ubiquitous genes. To be useful for normalization, a gene needs to be expressed in as many samples as possible and yet avoid extreme expression values. The first stage in our algorithm identifies ubiquitous genes (using 5% trimming) and retains them for further consideration as potential guide genes.

A

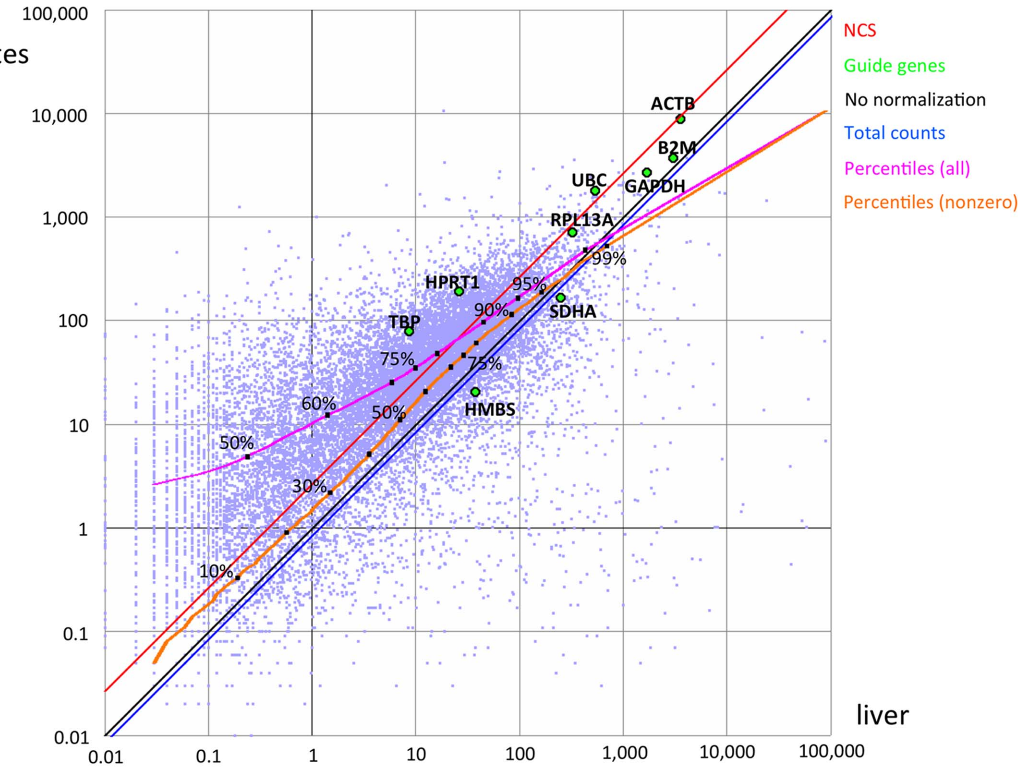
testes
liver
NCS
Guide genes
No normalization
Total counts
Percentiles (all)
Percentiles (nonzero)
ACTB
B2M
UBC
GAPDH
RPL13A
HPRT1
TBP
SDHA
HMBS
100,000
10,000
1,000
100
10
1
0.1
0.01
50%
60%
75%
90%
95%
99%
10%
30%
50%
75%

B

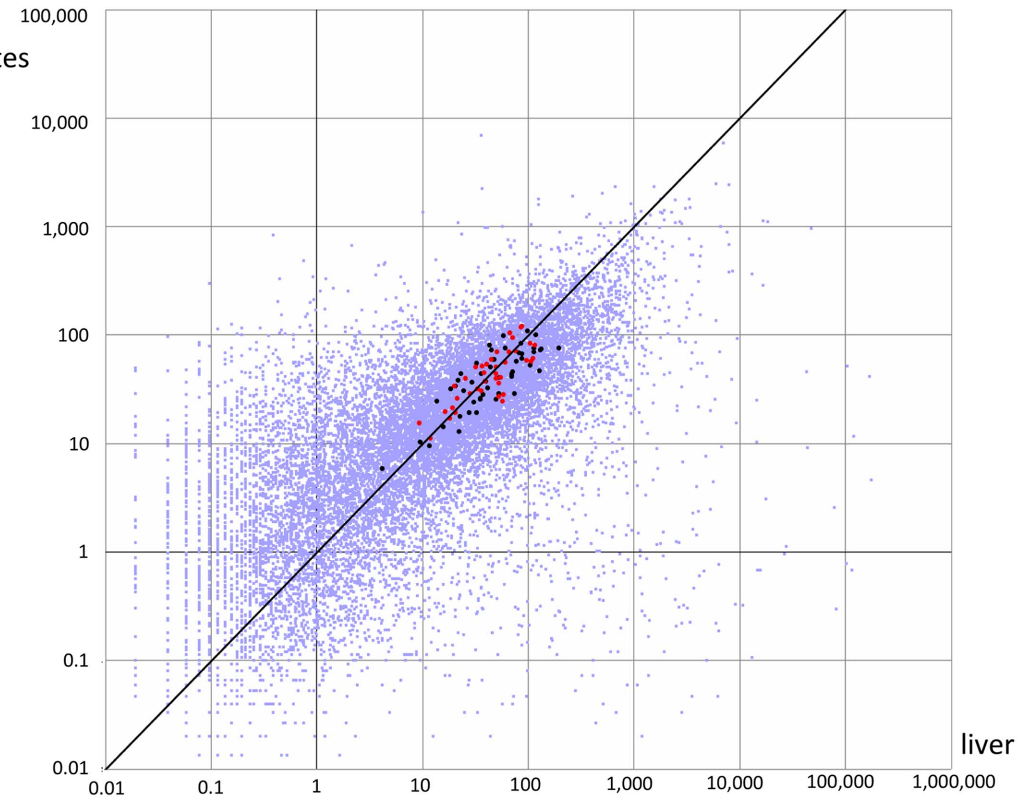
testes
liver
100,000
10,000
1,000
100
10
1
0.1
0.01
1,000,000

Figure 2. Pairwise comparison of expression levels. We compared the levels of expression of 15,861 genes with nonzero expression levels in both liver and testes, expressed in terms of average coverage per base. Each point represents one gene. A) Data prior to normalization. Housekeeping genes are highlighted as green points and labeled. The blue and red diagonals represent the relative correction factors computed based on total counts or the NCS method, relative to no normalization (black). The magenta and orange curves depict the percentiles when considering all genes or genes with nonzero values, respectively. B) Values after correction by NCS. Points in black or red denote genes with positive weights, and that therefore guided the scaling. Points in red denote the 39 genes with weight >0.5.
doi:10.1371/journal.pone.0077885.g002

Non-ubiquitous genes are temporarily ignored, but are considered again when doing the final scaling step. Depending on the data set, a large number of genes may be considered ubiquitous. Therefore, for efficiency purposes, one may use expression levels to sort the genes, and select an arbitrary number (e.g. 2000) of most highly expressed ubiquitous genes for further computation.

Stage 2: Pairwise comparison of ubiquitous genes. Appropriate guide genes are expected to be consistently proportional to each other across samples. The second stage in our algorithm performs all pairwise comparisons of expression levels between ubiquitous genes, and identifies *mutually consistent* gene pairs that display proportional levels of expression across samples. Let k be a sample and n be the number of samples studied; let Y_{gk} be the expression value of gene g in sample k. First we compute expression ratios $R_{ijk} = Y_{ik}/Y_{jk}$ for all pairs of ubiquitous genes i and j. Then, we identify the maximal and minimal ratios $max_{ij} = \text{max\_k}(R_{ijk})$ and $min_{ij} = \text{min\_k}(R_{ijk})$, and compute the ratio dispersal metric $D_{ij} = \log_2(max_{ij})\text{-}\log_2(min_{ij})$. Lower values of D_{ij} indicate genes with more mutually consistent expression levels: two genes expressed at identical levels, or at proportional levels, would achieve a dispersal value of zero. We discard gene pairs with $D_{ij} > 1$, i.e., for which the highest ratio of expression in a sample is over twice higher than the lowest ratio of expression in another sample.

Stage 3: Network analysis. The ensemble of mutually consistent gene pairs can be modeled as a weighted, undirected network, with the most central genes being the most informative. For edge weights, we use the pairwise similarity metric between two genes i and j: $sim_{ij} = 1/(1+ D_{ij})$. This similarity metric ranges from zero (or 0.5, with our current D_{ij} cutoff) to one, with a value of one being achieved by gene pairs expressed at identical or perfectly proportional levels in all shared samples. We then use the PageRank algorithm [30] to identify the most central nodes in the gene expression similarity network. PageRank is the algorithm originally used in the Google search engine to identify web pages of interest based on web link patterns, and can be applied as a general tool for computing node centrality in a network. The algorithm starts by giving all nodes equal values, and then iteratively redistributes the node values according to the linkage structure and edge weights. Upon convergence, the most central nodes have the highest PageRank value, at the expense of more peripheral nodes. Finally, we transform the PageRank values into gene-specific weights W_g defined as $W_g = PageRank_g \bullet G$ - 1 where G is the number of nodes (genes) included in the network. Negative weights are set to zero and discarded.

Stage 4: Computation of sample-specific scaling factors. Each gene can be used to suggest individual scaling factors for each sample, and the factors suggested by several genes can be combined, taking gene weights into account. This stage computes a scaling factor per sample (f_k), which satisfy $\sum_g (W_g \cdot \log_2(Y_{gk} \cdot f_k)) = C$, where C is an arbitrary value, constant across samples.

Stage 5: Data scaling. Having computed the scaling factors for each sample, this stage simply performs the final scaling, which renders gene expression levels comparable across samples, by multiplying each Y_{gk} by the corresponding f_k.

Solution Optimization via Evolution Strategy

We implemented a randomized Evolution Strategy (ES) algorithm [31] aiming to maximize the number of uniform genes. For each individual solution in the ES, the object parameter vector **y** corresponds to a vector of scaling factors, one factor per sample in the data set. The individual's observed fitness F(**y**) equals the objective function of normalization success, namely the number of uniform genes observed after scaling the raw data using the scaling factors specified by **y**.

We create an initial population of solutions, either randomly generating 10 individual solutions, or based on a user-specified set of previously computed solutions. The population is then grown, sorted and trimmed in rounds. At each round, we generate and add to the population: a) 5 mutant offspring of the highest-ranked individual so far, each offspring modifying each value in the parameter vector by a small, randomly picked value, b) 5 mutant offspring of randomly picked parents from individuals ranked #2 through #10, c) 5 mutant offspring of randomly picked parents from the remaining individuals in the population, d) 5 offspring produced by recombining two different, randomly picked individuals.

Finally, all individual solutions (including parents and offspring) are sorted by decreasing fitness, and the population is limited to the 200 top individuals. The process is repeated until either a) the system reaches convergence, as determined by no further increase in the fitness of the highest ranked individual for 100 consecutive rounds, or b) a user-specified amount of total time has elapsed.

Implementation and Availability

We have encapsulated the various normalizing methods into an easy to use Perl module, "Normalizer.pm". The module is built in a "cascade of functions" format, embodying the pipeline of the method. Each function in the pipeline automatically executes the function that precedes it if its results are not yet available. This means that a script can simply instantiate a new Normalizer object, specify the location of the data set, and call the module's *normalize* function directly. This will trigger execution of the entire analysis pipeline. Alternatively, one could set the data, specify gene weights (e.g. listing housekeeping genes), and use the *normalize* function to achieve the normalization based on the selected genes. Therefore the module can be used to perform different types of normalizations. Intermediate results are optionally stored on disk for efficiency, making it easier to test different parameters for each step in the pipeline. To compute PageRank, we use the Graph::Centrality::PageRank v. 1.05 Perl module by Jeff Kubina (http://search.cpan.org/~kubina/Graph-Centrality-Pagerank-1.05/lib/Graph/Centrality/Pagerank.pm), and Graph v. 0.94 by Jarkko Hietaniemi (http://search.cpan.org/~jhi/Graph-0.94/lib/Graph.pod). Our Normalizer.pm module implements several normalization algorithms (see Methods) and can be used flexibly to normalize data by one or more methods, and to evaluate the results.

In addition to the Perl module, we provide a simple command-line tool for normalizing data sets. The tool is a short Perl script that uses the Normalizer.pm module. This tool is distributed together with the Normalizer code, to serve both as a simple method to use the code, and as an example of how to develop code that uses the Normalizer.

All the software has been released as open source (GNU General Public License) and is available at http://db.systemsbiology.net/gestalt/normalizer/. The Perl code was also deposited in GitHub at https://github.com/gglusman/Normalizer.

Results

A Conceptual Taxonomy of Scaling Methods

We studied a wide variety of normalization algorithms, some published and some of our own devising (see Methods), and identified a small number of core concepts on which they are based. With the single exception of the Quantile Normalization method [18], all the algorithms we considered are global procedures that scale all the values in each sample using a single scaling factor [11,16]: we present their conceptual taxonomy in Fig. 1.

Some of the scaling methods are *based on a global characteristic of each sample* (Fig. 1, left), i.e., they study each sample independently and identify a characteristic value used for normalization. This characteristic value can be the sum of all counts (CPM method) [13], or a specific expression level, e.g., at the upper quartile [16]. We added two variations on these methods: scaling each sample's total count to the average count per sample ("Total" method), and scaling to the upper decile.

Alternatively, some scaling methods are *based on pre-selected genes* (Fig. 1, right), either using the expression value of a single housekeeping gene to guide normalization [14], or selecting the subset of housekeeping genes that are most consistent with each other ("most stable") and using the geometric mean of their expression values to guide normalization (geNorm method) [22].

Methods of the third class, which are *based on genes selected from the data* (Fig. 1, bottom), start by identifying a (usually large) set of genes expressed in the samples to be compared, and then use various combinations of these genes to derive the scaling factors that render the samples comparable. The TMM algorithm [12] is one such fully data-driven method, based on pairwise sample comparison of "double-trimmed" genes (i.e., trimmed first by absolute expression level ranks within each sample, and then by expression ratios between the two samples). We explored a variety of novel methods that use single trimming (by expression level ranks within each sample) and that scale all samples simultaneously. In particular, we created the novel Network Centrality Scaling (NCS) algorithm that uses pairwise correlation of gene expression levels as a similarity metric, and identifies the most central genes in the resulting network. These central genes are then used as normalization guides. We also implemented methods that select random sets of genes to serve as controls for the methods that choose gene subsets rationally.

Finally, scaling methods can be devised *using randomly picked values* (Fig. 1, top). In particular, we implemented an Evolution Strategy algorithm that stochastically identifies solutions that maximize, as objective function, the number of genes expressed uniformly across samples.

Computational Dissection of Normalization Methods

Even when based on very different concepts, the various normalization methods may share one or more computational procedures in common. We dissected the methods into distinct computational steps and identified shared components among the various algorithms (Fig. S1). There are four different initial actions: 1) ignore the data except for the pre-determined housekeeping genes; 2) ignore the data entirely and select random scaling values; 3) use the data solely to compute the total expression in each sample; 4) sort the data matrix in preparation for a variety of more complex computations. The sorted data matrix can then be used to compute rank-specific averages (for the Quantile Normalization method), to identify the expression levels at different percentiles of the distribution (for the upper quartile and upper decile methods), or to identify ubiquitous genes (see Methods). Ubiquitous genes are then used by several methods in diverse ways.

We identified three different possible endpoints (Fig. S1). The Quantile Normalization method produces a set of jointly normalized distributions. The CPM method produces absolutely scaled values. All the algorithms we describe here yield a set of relative scaling factors, which we adjust to keep the global scale of the data set (see Methods). These scaling factors can be computed from: 1) equal gene weights, 2) variable gene weights, 3) whole-sample weighted means, 4) a target value per sample, or 5) random values.

We found that best results are obtained with methods involving stochastic optimization, and with certain methods based on analysis of ubiquitous genes.

Application to a Real Dataset

We used the Illumina Bodymap 2.0 data set to compare the expression levels of 29,665 genes across a panel of 16 tissues; for each gene in each sample, we computed the expression level in terms of average coverage per base (see Methods). We then applied a series of normalization methods to this expression matrix. The normalization algorithms are described in the Methods.

Since expression levels may span several orders of magnitude, the comparison of gene expression levels in two samples may be visualized in a log-log scatterplot; as an example, we show the pairwise comparison of the expression levels of 15,861 genes observed in both liver and testes (Fig. 2). The effect of the various scaling methods considered here (all methods except for Quantile Normalization) is to shift the plot in one direction, without distortion: multiplying all observed values by a scaling factor is mathematically equivalent to a uniform shift in logarithmic scale. As expected, the expression levels are largely correlated, but the bulk of the point cloud is shifted to one side of the diagonal (black). While some of this shift is due to differences in gene expression levels among the two samples, the main effect is due to different sequencing depth, and different allocations of reads among different genes, in each of the two samples. Without any normalization, 9,972 genes were observed in testes at coverage at least double that observed in liver, and conversely just 1,516 genes were expressed in liver at least twice as strongly as in testes. Only two genes (*PTGDS* and *PRM2*) were observed in testes at coverage slightly higher than 10,000/base, compared to 13 genes in liver, some of which exceeded coverage of 50,000/base (*ALB, HP, SAA1, FGB, SERPINA1, CRP*).

After scaling to CPM or Total counts (see Methods), the expression levels of 10,837 genes in testes were higher than twice their liver values, but the reverse relation only held for 1,516 genes; 15 genes had coverage higher than 10,000/base in liver, but none in testes. Therefore, the simple normalization based on the total number of reads in each sample (blue diagonal) only increased the skews observed in the expression values prior to normalization, demonstrating the main flaw of this method.

We next considered scaling guided by individual housekeeping genes (large green circles in Fig. 2A), for example the ubiquitous and abundant *GAPDH* gene. This correction decreased the skew between the liver and testes samples. Nevertheless, many more genes still displayed an apparent higher expression level in testes than in liver (7,468 and 2,369 for testes and liver, respectively, at or above the 2-fold cutoff). While either *RPL13A* or *ACTB* could

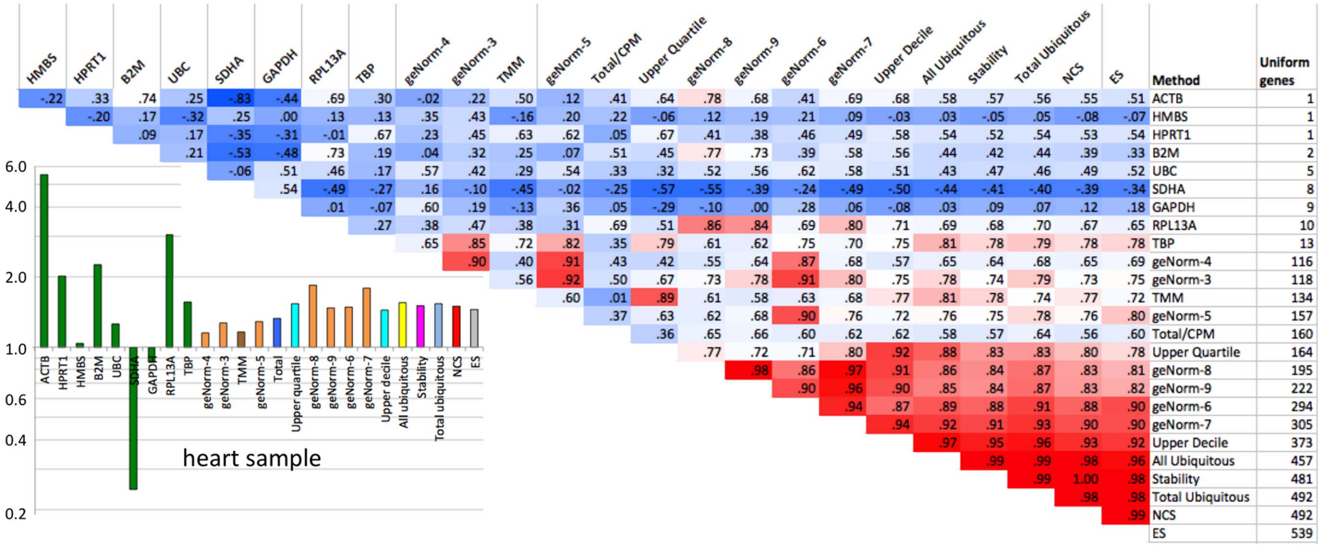

Figure 3. Comparison between the scaling factors suggested by the different methods. Lower left: the resulting scaling factors for the heart sample. Upper right: Pairwise correlations between the methods, for all samples. Red shades denote high correlation values (above 0.75), blue denotes low correlation (or anticorrelation). The column to the right indicates the number of uniform genes identified by the method. The Quantile Normalization method is not included in this analysis since it does not produce scaling factors.
doi:10.1371/journal.pone.0077885.g003

have served as successful individual guide genes in this specific two-tissue comparison, they did not perform well when comparing other samples, e.g., skeletal muscle vs. lymph node (Fig. S2).

Scaling to the level of expression at a given percentile (e.g., median value, upper quartile, upper decile) was very sensitive to the precise cutoff used (magenta curve in Fig. 2A). Scaling to a percentile after exclusion of nonzero values yielded much more consistent results across possible cutoffs up to the upper decile (orange curve in Fig. 2A), but was consistently skewed away from the center of the point cloud, and towards highly expressed genes.

Scaling the liver vs. testes expression values via the NCS algorithm (Fig. 2A, red line; levels after scaling are shown in Fig. 2B) corresponds to similar expression of most genes in these tissues at all expression levels, and yields similar numbers of genes more highly expressed in either sample (4,628 testes >2x liver, 4,373 liver >2x testes). The NCS method finds and exploits genes (89 for this data set, Fig. 2B; red points, centrality weight >0.5; black points, centrality weight <0.5) that are central in a graph representing similarity of expression level between genes. This network property corresponds to "depth" within the point cloud. The ten most central genes were *XPO7, TNKS2, AMBRA1, C4orf41, C17orf85, GLG1, CCAR1, EXOC1, RBBP6* and *WDR33*. While none of these genes are usually considered "housekeeping" genes, most function in basic cellular maintenance processes, and *CCAR1*, *GLG1* and *EXOC1* were previously recognized as candidate housekeeping genes [32].

We next compared the scaling factors for each sample in the set, as suggested by several scaling methods. In every case we observe discrepant results, as exemplified by the heart sample (Fig. 3, lower left). Scalings based on single housekeeping genes usually yield the most extreme scaling factors and are most frequently at odds with most other methods. The "total counts" method usually (but not always) agrees with other methods in the direction of change needed, but its magnitude is strongly affected by highly expressed tissue-specific genes.

We considered whether the solutions offered by the different algorithms (and yielding varying numbers of uniform genes) are substantially different. To assess this, we computed all pairwise correlations between the vector of scaling factors produced by the various algorithms (Fig. 3, upper right). This computation confirms that scalings based on single housekeeping genes are significantly different from each other and from other solutions; scaling based on the *SDHA* gene is frequently anti-correlated with most other solutions. On the other hand, several independent methods produce very similar solutions, as reflected by the high correlation between their respective vectors of scaling factors. We show next that these solutions are also among the most successful, as assessed by two different metrics: 1) ability to identify uniformly expressed genes, and 2) decorrelation of sample ranking.

Quantifying Success by Identifying Uniform Genes

The first metric is based on the analysis of gene-specific coefficients of variation (CoV). The average CoV can be used to assess the level of dispersion in the data: a lower average CoV reflects stronger reduction of technical noise [18]. This measure is sensitive to outlier high CoV values, which is expected of sample-specific genes. Additionally, the distribution of CoV values is long-tailed, and its shape depends on the normalization (Fig. S3). We therefore concluded that the *average* CoV is not a suitable measure of normalization success. Nevertheless, the CoV of an individual gene is a valuable metric for quantifying how successful the normalization was at rendering its expression values consistent across samples.

We defined a gene as *uniformly expressed* (or simply "uniform") if the CoV of its post-normalization expression levels across all samples is less than a low cutoff, arbitrarily but judiciously chosen to be strict and to maximize the separation between the various methods (Fig. S4). The absolute number of "uniform" genes thus identified will strongly depend on the cutoff value, but the relative values among different normalization methods are quite stable, yielding similar results for a variety of possible cutoff values (Fig. S5). We reasoned that methods that are more successful at equalizing gene expression values across samples would yield larger numbers of uniform genes. The number of uniform genes as defined here is largely unaffected by the presence of sample-

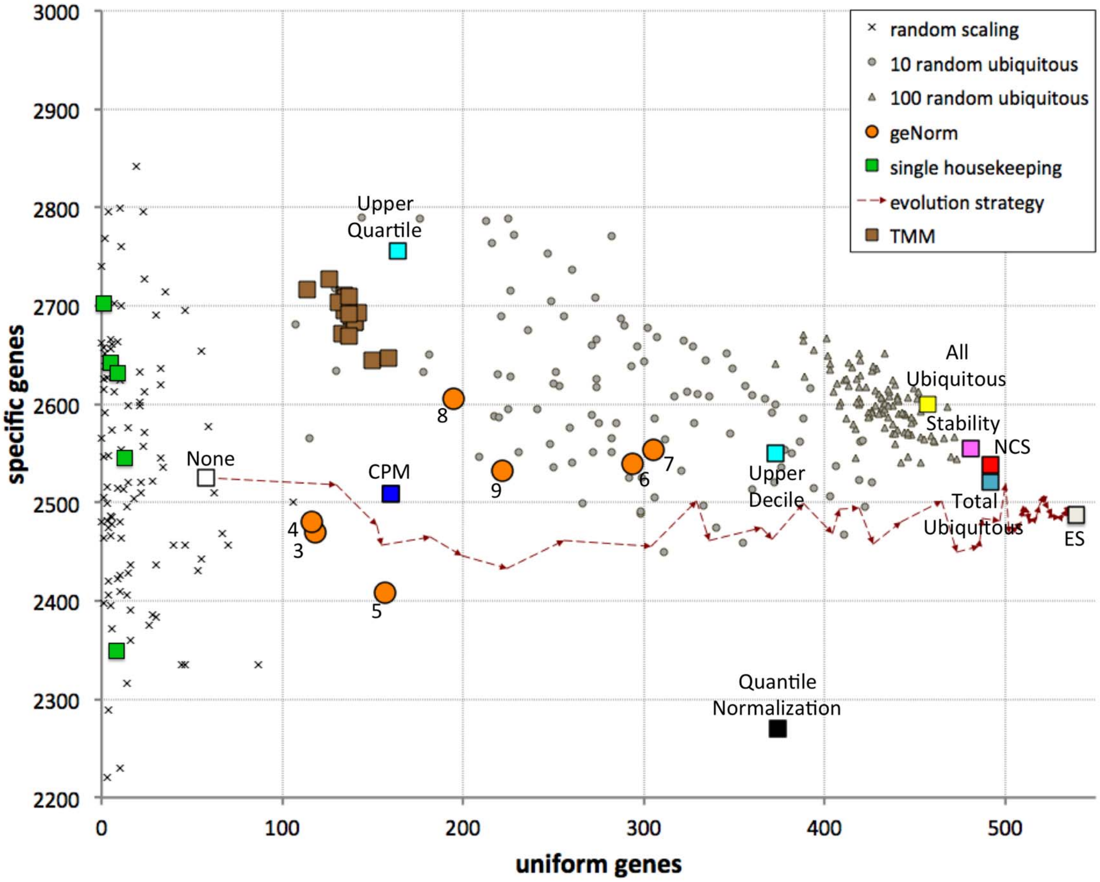

Figure 4. Comparison of performance of various normalization methods. Each method is evaluated by the number of genes observed to be consistently expressed across samples (abscissa); different methods also yield different numbers of genes identified as specific to one sample. The numbers in the orange circles denote the number of housekeeping genes combined using the geNorm algorithm. The dashed arrows show one stochastic path of the ES from the data prior to normalization (white square, "None") to the best approximation to the optimal solution (gray square, ES). Brown squares represent the results obtained via the TMM method, using each of the 16 samples as reference.
doi:10.1371/journal.pone.0077885.g004

specific genes, and is therefore a more stable metric of normalization success than the CoV.

Using these definitions and a gene CoV cutoff of 0.25, we observed 58 uniform genes and 2,525 specific genes in the data prior to normalization. We explored the performance of the different normalization methods by comparing the number of uniform genes and the number of specific genes after normalization (Fig. 4). Most (but not all) scaling methods significantly increase the number of uniform genes, as expected and desired for successfully normalized data, with more modest changes in the number of specific genes. In contrast, mock normalization in which samples were scaled by a randomly picked factor resulted in far fewer uniform genes and frequently created many more "specific" genes.

Scaling based on single housekeeping genes identified a surprisingly small number of uniform genes, in a range similar to the random scaling (13 with *TBP*, 10 with *RPL13A*, 9 with *GAPDH*, etc.). In particular, scaling based on three of the housekeeping genes tested (*ACTB*, *HPRT1* and *HMBS*) failed to render any other genes uniform. This demonstrates that single housekeeping genes are not appropriate guides for normalizing this data set. On the other hand, geometric averaging of several housekeeping genes identified many more uniform genes (geNorm method, orange circles in Fig. 4); best results (305 uniform genes) were achieved by combining seven housekeeping genes (*UBC, HMBS, TBP, GAPDH, HPRT1, RPL13A* and *ACTB*).

Scaling to total expression (CPM), to the upper quartile, or via the TMM method was only moderately successful at producing uniform genes; many more uniform genes were produced by scaling to the upper decile (373 genes). The expression levels normalized by the non-proportional Quantile Normalization method identified 374 uniform genes, but a much reduced number of specific genes; this is consistent with the variance loss inherent in this method. The very simple scaling to the total expression in ubiquitous genes outperformed all these methods, yielding 492 uniform genes.

While the simple mock normalization by randomly picked scaling factors is strong enough a control for assessing single housekeeping genes, it is a weak negative control for more complex algorithms. Most of the algorithms we consider are based on selecting a set of genes and combining them to produce scaling factors; the number of genes is up to 10 for some methods (e.g., geNorm) and ~100 for others (e.g., stability, NCS). We therefore implemented a stronger negative control in which we select 10 or 100 randomly picked ubiquitous genes, and use them to guide normalization (gray circles and triangles in Fig. 4), or simply use all ubiquitous genes (yellow square). We find that scaling based on 100 randomly picked ubiquitous genes, or all ubiquitous genes, always produces superior results to those attained by the CPM,

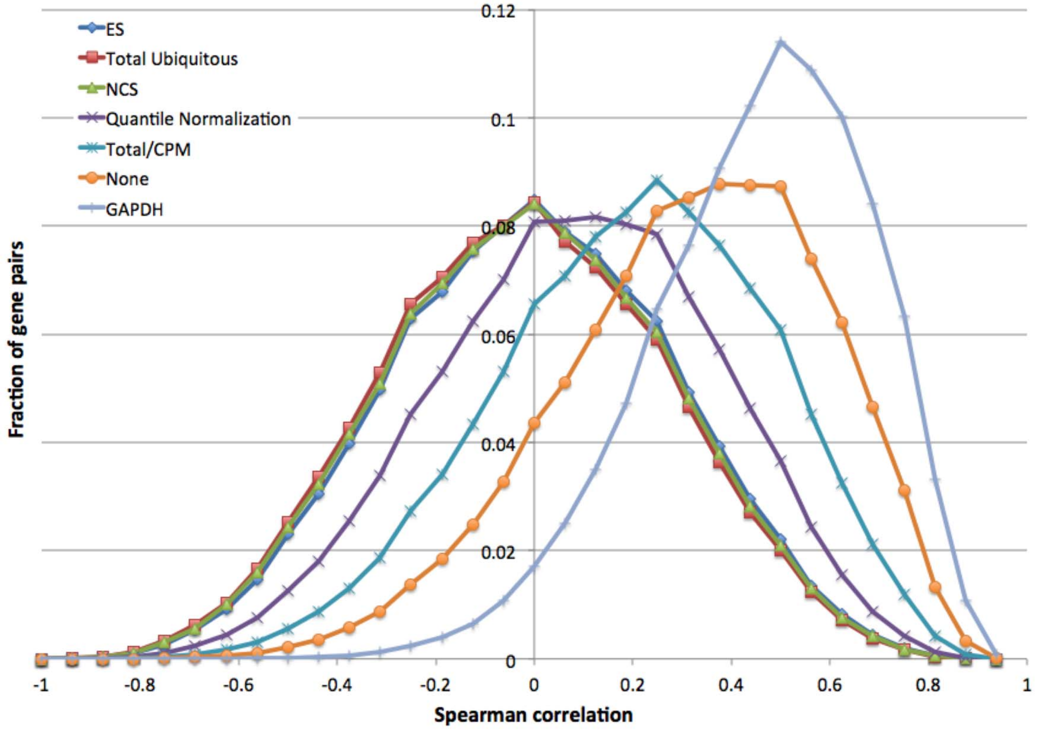

Figure 5. Density distribution of Spearman correlations of sample rankings for some normalization methods.
doi:10.1371/journal.pone.0077885.g005

upper quartile, upper decile, Quantile Normalization, TMM and geNorm methods. The success level of scaling via a smaller set of 10 randomly selected ubiquitous genes is much less consistent, but is almost always superior to CPM, upper quartile and TMM normalization.

We note that certain random subsets of 100 ubiquitous genes outperform the results of the entire set of ubiquitous genes. This indicates that there is opportunity for improved success by algorithms that judiciously select among the ubiquitous genes to be used to guide normalization. Indeed, applying the "gene stability" metric [22] to select 100 guides from the entire set of ubiquitous genes yielded excellent results (481 uniform genes), again highlighting the inadequacy of small sets of pre-selected housekeeping genes for guiding normalization efforts. Similarly, using gene network centrality to select guide genes (i.e., the NCS method) identified 492 uniform genes (Fig. 4).

The availability of an objective function (number of uniform genes) for quantifying the success level of normalization methods provides a unique opportunity: it is now possible to use a variety of optimization algorithms to search the solution space of possible scaling normalizations, aiming to identify solutions maximizing the objective function. We considered using traditional gradient-based optimization algorithms, but rejected this option since the "fitness terrain" of this objective function is not smooth and presents many local maxima (Fig. S6). We therefore decided to implement a stochastic Evolution Strategy (ES) algorithm to search for high-scoring sets of scaling factors. Starting from no normalization (all factors equal to 1), the ES 1) generates solutions by a variety of deterministic and random methods, 2) selects the most successful among these solutions, and 3) iterates this process and progressively identifies solutions with increased fitness relative to those observed until that moment. By analyzing intermediate results at regular intervals, it is possible to visualize the progress of the ES run, as shown by the dashed arrows in Fig. 4. The ES gradually produces solutions as good as those identified by the other methods, and eventually surpasses them. In our current example (Illumina BodyMap 2.0), the ES identified a set of sample-specific scaling factors that bring 539 genes to uniform expression levels, while identifying 2,487 sample-specific genes (rightmost point in Fig. 4). Repeated runs of the ES consistently produced results with over 530 uniform genes.

Quantifying Success by Decorrelation Analysis

The second metric, inspired by the gene decorrelation analysis of Shmulevich [11], measures the mutual information content of gene pairs. In short, we first ranked the samples independently for each gene, according to gene expression values; we then computed the Spearman correlation of these rankings for gene pairs, and assessed the global mutual information content in the expression matrix by the average correlation (see Methods). Consider the two-sample comparison in Fig. 2. By expression level prior to normalization (Fig. 2A), the vast majority of the genes ranked the testes sample over the liver sample. Similarly skewed relations were apparent for other sample pairs (e.g., Fig. S2). Therefore, the sample ranking correlations were high for most gene pairs. Unsuccessful normalization methods increased sample ranking correlations, shifting the distribution further to the right (Fig. 5).

In contrast, after successful normalization (e.g., Fig. 2B) many gene pairs disagreed on sample ranking, leading to lower average correlation. We found that the most successful normalization methods (ES, NCS, Total ubiquitous) yield symmetric correlation distributions centered around zero (Fig. 5), suggesting these methods are approaching the optimal solution.

Discussion

Data standardization is a crucial first step in the analysis of RNA-seq and other digital transcriptome data. In particular, any

attempt to identify differential expression requires careful scaling of the data to make different samples comparable. Distorted results may produce both false leads, suggesting differential expression due to incorrect upscaling, and missed opportunities, when true differential expression is masked by improperly downscaled values.

We have developed novel data-driven scaling algorithms that use combinations of ubiquitously expressed genes as the basis to compute sample-specific scaling factors. These sample-specific scaling factors are correct only in the context of their comparison to other samples: the same sample may be scaled differently depending on what other samples it is being compared to. This stands in contrast with the absolute scaling factors computed via the CPM method.

We applied these and other algorithms to study sets of human RNA-seq data. The results revealed very significant distortions in gene expression values as normalized by simpler methods, consistent with previous reports [33]. For the very commonly used CPM normalization method, these distortions can be traced to the presence of very highly expressed tissue-specific genes. The other commonly used method, which relies on individual "housekeeping" genes presumed to be expressed at constant levels, also leads to significant distortions and to misdirected conclusions about tissue specificity of genes. While judicious combinations of housekeeping genes yield more reliable results, we found that these can still be much improved.

We analyzed the Illumina BodyMap data sets, which include samples from a variety of normal human tissues. We also applied the analysis methods to RNA-seq data sets including sample replicates (not shown). The CPM/Total methods perform significantly better when analyzing data sets dominated by replicates (technical or biological) from the same tissues than when analyzing samples from diverse tissues. This is the expected result since replicates are not expected to include sample-specific genes. The presence of replicates may also affect the decorrelation analysis: in a data set dominated by sample replicates (technical or biological), all correlation values may be inflated relative to more diverse data sets. For a given data set, optimal scaling will nevertheless minimize correlation.

The effectiveness of normalization methods is typically measured in terms of their ability to identify differentially expressed genes [33]. We proposed a novel approach for evaluating the success of normalization algorithms, based on their ability to identify "uniform" genes. The set of broadly expressed genes can be several thousand strong [32]. We further focus on the subset of genes whose expression pattern is not only broad and defined as "housekeeping" *a priori*, but are also essentially invariant across samples based on examination of the dataset of interest. The biological hypothesis behind this approach is that, while different cell types have their characteristic patterns of expression of specific genes, there exists a large "core" of genes that are expressed in a consistent pattern across cell types. The alternative hypothesis is that in different tissues most genes are differentially regulated, and that such "core" of uniformly expressed genes does not exist, or is very limited. In this case, the expectation is for the set of computed uniform genes to be small and unstable, strongly depending on the specific sample scaling factors used. Our results confirm that it is possible to identify a large set of genes that are consistently expressed across samples representing disparate tissues. Stochastic sampling via the Evolutionary Strategy algorithm robustly converged on nearly identical sets of uniform genes. Moreover, this set of genes is similar to those identified as uniform after scaling using the geNorm, Upper Quartile and Upper Decile algorithms - neither of which relies on precomputation of uniform genes.

Previous evaluation methods have been based on the concept of reducing variation of gene expression across samples, but have either focused on very small sets of pre-selected genes, or considered the global variation of all genes in the sample. We find that the former uses only a small fraction of the available information in the data, and the latter is inappropriate due to the long-tailed distribution of gene expression variations (Fig. S3). Our approach takes advantage of the expression patterns of hundreds of genes, which we directly select from the data, avoiding such distortions.

The total amount of RNA produced by a cell may have significant implications on cellular size and metabolism – a biological fact that data-driven normalization methods may fail to model [15]. Normalization based on the core of "uniform" genes will have this same deficiency if most core genes are expressed at a proportionally higher or lower rate. Nevertheless, these methods should detect deviations from such proportionality.

Our Network Centrality Scaling method is based on the PageRank algorithm [30], which uses the link structure of the Web to compute a web page's authority and hence rank search results. The concepts and methodology behind PageRank have been fruitfully used in other contexts, e.g., ranking proteins based on the similarity network [34] and identifying genes responsible for adverse drug reactions [35]. In our implementation, the links in the network of genes represent the extent to which their expression patterns are mutually consistent. Thus, in similarity with social network analysis, in which the number, strength and topology of interpersonal links can define popularity, the number, strength and topology of coexpression links identifies the most "central" genes in the expression network. We proposed and verified that these genes, weighted by their centrality, can serve as effective scaling guides.

In addition to the deterministic methods based on analysis of ubiquitously expressed genes, we implemented a stochastic Evolutionary Strategy search for solutions that maximize the number of uniform genes across samples. This algorithm has two disadvantages. First, being non-deterministic, repeated runs produce slightly different results; there is no guarantee that the optimal solution will be reached in any particular run. While results from different runs are slightly different, they are nevertheless highly similar to each other. Second, the ES is computationally intensive, since it requires testing very large numbers of solutions. It takes much longer for the ES to converge on an optimal solution than the time required to compute any of the deterministic methods: typically in the order of a few hours for our current implementation, as opposed to a few seconds. On the other hand, there is room for optimization by parallelization, and normalization of sets of RNA-seq samples is not a performance bottleneck in current analysis pipelines. Therefore, these two deficiencies are not fatal.

An interesting result emerges from the implementation and application of a large number of algorithms to analyzing the same data set: while many of them produce disparate and incompatible results, several algorithms produce solutions that are strongly correlated (Fig. 5). We obtained similar solutions from seven disparate algorithms: ES, NCS, Stability, Total Ubiquitous, Upper Decile, All Ubiquitous, and the combinations of the geNorm method that rely on several housekeeping genes. These algorithms are based on many different concepts and involve very different computational procedures (Fig. 1). Using the upper quartile algorithm, or random selections of 100 ubiquitous genes, also tend to produce similar results. Results from the TMM method are also reasonably well correlated with those of the seven algorithms. Of all the single housekeeping genes we considered, only *TBP* yielded

a scaling solution that is somewhat similar to all these methods. Importantly, there was no other, separate cluster of solutions from independent methods. This suggests that an optimal solution to the scaling problem exists, and that some of the algorithms independently approach it.

One characteristic of this optimal solution is that it presents the largest number of uniform genes, a result intuitively expected from successful normalization. We stress that, with the sole exception of the ES, the identification of uniform genes is not directly part of any of the algorithms described. The NCS algorithm and the Stability method ultimately rely on pairwise comparisons of gene expression levels, not on homogenization of individual genes. Finally, the Total Ubiquitous method combines the expression levels of several thousand genes by simple addition, without attempting to homogenize the expression level of any one of them, and yet it reaches a similar scaling solution.

Another characteristic of the optimal solution is that it fully decorrelates sample ranking as assessed by comparing gene pairs, a result again expected for successful normalizations. We stress that this metric is conceptually and computationally independent from the identification of uniform genes, and that it was not explicitly optimized by any of the algorithms.

Based on the combined evidence from these three independent considerations (similarity of results, maximization of uniform genes, and maximal decorrelation), we conclude that the ES algorithm provides the best approximation to the optimal solution to the scaling task. The Total Ubiquitous, NCS and Stability algorithms are significantly faster, and reach very similar solutions in a deterministic fashion. In situations where computation speed is a prime consideration, we recommend using these methods to achieve the fastest approximation to the optimal solution.

We expect these novel approaches to provide accurate and robustly scaled expression values, leading to improvements in the identification of differentially expressed and tissue-specific genes.

Supporting Information

Figure S1 "Bus map" graph of normalization algorithms. Each normalization method is represented by a path from the raw data matrix (green rounded square) to one of three types of outcomes (red rounded squares). Open rounded rectangle "stations" denote intermediary results. Large rectangles with blue background label the first unique step of each method. Non-deterministic methods are indicated with dotted or dashed lines.
(TIFF)

Figure S2 Comparison of expression levels of 12834 genes with nonzero expression levels in both skeletal muscle and lymph node, expressed in terms of average coverage per base. Each point represents one gene. Housekeeping genes are highlighted as green points and labeled. The blue and red diagonals represent the relative correction factors computed based on total counts or the NCS method, relative to no normalization (black). The magenta and orange curves depict the percentiles when considering all genes or genes with nonzero values, respectively.
(TIFF)

Figure S3 Distribution of gene Coefficients of Variation after normalization based on several methods.
(TIFF)

Figure S4 Cumulative distribution of gene Coefficients of Variation after normalization based on several methods.
(TIFF)

Figure S5 Specific vs. uniform genes, by several normalization methods, using four different Coefficient of Variance (CoV) cutoffs for defining genes as uniform. Graphics used are the same as in Fig. 4. Too few uniform genes are identified using a too-stringent cutoff (0.15). Conversely, using a too-lax cutoff (0.5) leads to identification of several thousand uniform genes by all methods, losing separation among the methods. Intermediate cutoffs (0.25–0.3) are most effective at distinguishing between different normalization methods.
(TIFF)

Figure S6 Fitness terrain when modifying the scaling factor for a single sample (liver) relative to the best solution found via the ES algorithm (539 uniform genes, using liver scaling factor of 1.6978). The upper-left and lower-left insets show increasing detail; the red portions of the curves indicate the range expanded in the subsequent graph. Several local maxima are observed. The ES solution (red arrow) falls within the global maximum range observed (liver scaling factors 1.6954–1.6988).
(TIFF)

Figure S7 Fitness terrain of the Total Ubiquitous method, testing all possible combinations of lower and upper cutoffs at 5% resolution. Numbers represent the resulting uniform genes; red and black backgrounds highlight increasingly successful combinations. The maximal values observed are highlighted in white font.
(TIFF)

Acknowledgments

We thank Gary P. Schroth and Illumina, Inc. for providing the RNA-seq samples. We thank Lee Rowen, Irit Rubin and Ilya Shmulevich for helpful discussions.

Author Contributions

Conceived and designed the experiments: GG LH. Analyzed the data: GG JC BK. Wrote the paper: GG JC MR.

References

1. Marioni JC, Mason CE, Mane SM, Stephens M, Gilad Y (2008) RNA-seq: an assessment of technical reproducibility and comparison with gene expression arrays. Genome Res 18: 1509–1517.
2. Velculescu VE, Zhang L, Vogelstein B, Kinzler KW (1995) Serial analysis of gene expression. Science 270: 484–487.
3. Brenner S, Johnson M, Bridgham J, Golda G, Lloyd DH, et al. (2000) Gene expression analysis by massively parallel signature sequencing (MPSS) on microbead arrays. Nat Biotechnol 18: 630–634.
4. Mortazavi A, Williams BA, McCue K, Schaeffer L, Wold B (2008) Mapping and quantifying mammalian transcriptomes by RNA-Seq. Nat Methods 5: 621–628.
5. Wang ET, Sandberg R, Luo S, Khrebtukova I, Zhang L, et al. (2008) Alternative isoform regulation in human tissue transcriptomes. Nature 456: 470–476.
6. Blekhman R, Marioni JC, Zumbo P, Stephens M, Gilad Y (2010) Sex-specific and lineage-specific alternative splicing in primates. Genome Res 20: 180–189.
7. Wu ZJ, Meyer CA, Choudhury S, Shipitsin M, Maruyama R, et al. (2010) Gene expression profiling of human breast tissue samples using SAGE-Seq. Genome Res 20: 1730–1739.
8. Lee S, Seo CH, Lim B, Yang JO, Oh J, et al. (2011) Accurate quantification of transcriptome from RNA-Seq data by effective length normalization. Nucleic Acids Res 39: e9.
9. Zheng W, Chung LM, Zhao H (2011) Bias detection and correction in RNA-Sequencing data. BMC Bioinformatics 12: 290.
10. Risso D, Schwartz K, Sherlock G, Dudoit S (2011) GC-content normalization for RNA-Seq data. BMC Bioinformatics 12: 480.

11. Shmulevich I, Zhang W (2002) Binary analysis and optimization-based normalization of gene expression data. Bioinformatics 18: 555–565.
12. Robinson M, Oshlack A (2010) A scaling normalization method for differential expression analysis of RNA-seq data. Genome Biol 11: R25.
13. Meyers BC, Tej SS, Vu TH, Haudenschild CD, Agrawal V, et al. (2004) The use of MPSS for whole-genome transcriptional analysis in Arabidopsis. Genome Res 14: 1641–1653.
14. de Kok JB, Roelofs RW, Giesendorf BA, Pennings JL, Waas ET, et al. (2005) Normalization of gene expression measurements in tumor tissues: comparison of 13 endogenous control genes. Lab Invest 85: 154–159.
15. Loven J, Orlando DA, Sigova AA, Lin CY, Rahl PB, et al. (2012) Revisiting global gene expression analysis. Cell 151: 476–482.
16. Bullard J, Purdom E, Hansen K, Dudoit S (2010) Evaluation of statistical methods for normalization and differential expression in mRNA-Seq experiments. BMC Bioinformatics 11: 94.
17. Bolstad B, Irizarry R, Astrand M, Speed T (2003) A comparison of normalization methods for high density oligonucleotide array data based on variance and bias. Bioinformatics 19: 185–193.
18. Mar J, Kimura Y, Schroder K, Irvine K, Hayashizaki Y, et al. (2009) Data-driven normalization strategies for high-throughput quantitative RT-PCR. BMC Bioinformatics 10: 110.
19. Qiu X, Wu H, Hu R (2013) The impact of quantile and rank normalization procedures on the testing power of gene differential expression analysis. BMC Bioinformatics 14: 124.
20. Hansen KD, Irizarry RA, Wu Z (2012) Removing technical variability in RNA-seq data using conditional quantile normalization. Biostatistics 13: 204–216.
21. Zhou L, Lim QE, Wan G, Too HP (2010) Normalization with genes encoding ribosomal proteins but not GAPDH provides an accurate quantification of gene expressions in neuronal differentiation of PC12 cells. BMC Genomics 11: 75.
22. Vandesompele J, De Preter K, Pattyn F, Poppe B, Van Roy N, et al. (2002) Accurate normalization of real-time quantitative RT-PCR data by geometric averaging of multiple internal control genes. Genome Biol 3: RESEARCH0034.
23. Hartemink AJ, Gifford DK, Jaakkola TS, Young RA (2001) Maximum likelihood estimation of optimal scaling factors for expression array normalization. SPIE BIOS 2001, San Jose, California, January 2001.
24. Sîrbu A, Ruskin HJ, Crane M (2010) Cross-Platform Microarray Data Normalisation for Regulatory Network Inference. PLoS ONE 5: e13822.
25. Kent WJ (2002) BLAT–the BLAST-like alignment tool. Genome Res 12: 656–664.
26. Trapnell C, Pachter L, Salzberg SL (2009) TopHat: discovering splice junctions with RNA-Seq. Bioinformatics 25: 1105–1111.
27. Trapnell C, Williams BA, Pertea G, Mortazavi A, Kwan G, et al. (2010) Transcript assembly and quantification by RNA-Seq reveals unannotated transcripts and isoform switching during cell differentiation. Nat Biotechnol 28: 511–515.
28. Kadota K, Nishiyama T, Shimizu K (2012) A normalization strategy for comparing tag count data. Algorithms Mol Biol 7: 5.
29. Jongeneel CV, Delorenzi M, Iseli C, Zhou D, Haudenschild CD, et al. (2005) An atlas of human gene expression from massively parallel signature sequencing (MPSS). Genome Res 15: 1007–1014.
30. Brin S, Page L (1998) The anatomy of a large-scale hyper-textual Web search engine. Computer Networks and ISDN Systems 30: 107–117.
31. Beyer H-G, Schwefel H-P (2002) Evolution strategies - A comprehensive introduction. Natural Computing 1: 3–52.
32. Zhu J, He F, Song S, Wang J, Yu J (2008) How many human genes can be defined as housekeeping with current expression data? BMC Genomics 9: 172.
33. Dillies MA, Rau A, Aubert J, Hennequet-Antier C, Jeanmougin M, et al. (2012) A comprehensive evaluation of normalization methods for Illumina high-throughput RNA sequencing data analysis. Brief Bioinform.
34. Weston J, Elisseeff A, Zhou D, Leslie CS, Noble WS (2004) Protein ranking: from local to global structure in the protein similarity network. Proc Natl Acad Sci U S A 101: 6559–6563.
35. Yang L, Xu L, He L (2009) A CitationRank algorithm inheriting Google technology designed to highlight genes responsible for serious adverse drug reaction. Bioinformatics 25: 2244–2250.
